# Genome-wide identification and expression pattern of SnRK gene family under several hormone treatments and its role in floral scent emission in *Hedychium coronarium*

**DOI:** 10.7717/peerj.10883

**Published:** 2021-03-10

**Authors:** Chutian Wang, Farhat Abbas, Yiwei Zhou, Yanguo Ke, Xinyue Li, Yuechong Yue, Yunyi Yu, Rangcai Yu, Yanping Fan

**Affiliations:** 1 The Research Center for Ornamental Plants, College of Forestry and Landscape Architecture, South China Agricultural University, Guangdong, China; 2 College of Life Sciences, South China Agricultural University, Guangdong, China; 3 College of Economics and Management, Kunming university, Kunming, China; 4 Guangdong Key Laboratory for Innovative Development and Utilization of Forest Plant Germplasm, South China Agricultural University, Guangdong, China

**Keywords:** *SnRK*, *Hedychium coronarium*, Hormones, Stress responses, Floral scent

## Abstract

The *SnRK* (Snf1-Related protein Kinase) gene family plays crucial roles in various plant signaling pathways and stress-adaptive responses including biotic and abiotic stresses via activating protein phosphorylation pathways. However, there is no information available on the role of the *SnRK* gene family in *Hedychium coronarium*. *H. coronarium* is an important crop widely cultivated as an ornamental plant, herb, spice, or condiment. In this study, 60 *HcSnRK* genes were identified from the *H. coronarium* genomic and transcriptome data. Phylogenetic and gene structure analysis showed that the *HcSnRK* genes were divided into three groups (*HcSnRK1*, *HcSnRK2* and *HcSnRK3*) and among them *HcSnRK3* subfamily was further subdivided into two clades according to the number of introns. Chromosome localization analysis showed that *HcSnRK* genes were unevenly mapped onto all chromosomes, and the *Ka/Ks* ratio of 24 paralogues includes four tandems and 20 segmental duplications indicated that the *HcSnRK* gene family underwent a purifying selection. *Cis*-regulatory elements analysis suggested that the *HcSnRK* genes respond to multiple hormones and other stresses. The responsiveness of *HcSnRK* genes to several hormones was analyzed by quantitative real-time PCR. Based on the different transcriptome data, two candidates *HcSnRK* genes (*HcSnRK2.2* and *HcSnRK2.9*) were screened out for further characterization . The subcellular localization experiment revealed that both genes were located in the nucleus and cytoplasm. Moreover, virus-induced gene silencing (VIGS) of *HcSnRK2.2* and *HcSnRK2.9* significantly reduced the floral volatile contents by suppressing the expression of terpene synthase genes (*HcTPS1, HcTPS3,* and *HcTPS5*), indicating that *HcSnRK2.2* and *HcSnRK2.9* genes play an important role in the regulatory mechanism of floral aroma. These results will provide novel insights into the functional dissection of *H. coronarium SnRK* gene family.

## Introduction

Floral scent is an important plant secondary metabolite that plays key roles in different developmental processes. Floral scent also plays a crucial role in plant communication both above and below-ground and stress signaling (Dudareva et al., 2006; Das et al., 2013; Muhlemann, Klempien & Dudareva, 2014; Abbas et al., 2017). The main role of floral scent is to protect the plant against external stimuli (biotic and abiotic stresses) and attract pollinators (Dudareva et al., 2006; Das et al., 2013). According to biosynthetic pathway, terpenoids, benzenoids/phenylpropanoid, and derivatives of fatty acids are the main classes of volatile organic compounds (VOCs) emitted from the plant surface. Among them, terpenoids constitute the largest class of plant secondary metabolites playing several functions throughout plant life and the expression profile of terpene synthase genes determines the involvement of terpenoid metabolites in different ecological and physiological functions in response to external stimuli. Protein kinases are considered as central components in defense mechanisms, which activate several protein phosphorylation pathways to regulate the expression of downstream genes related to stress response (Tena, Boudsocq & Sheen, 2011). In recent years, protein kinases such as mitogen-activated protein kinase (MAPK) (Xu & Zhang, 2015), calcium-dependent protein kinases (CDPK) (Baba, Rigó & Ayaydin, 2018), glycogen synthase kinase 3 (GSK3) (Beurel, Grieco & Jope, 2015), and sucrose non-fermenting 1 (SNF1) related protein kinases (SnRK) (Hrabak et al., 2003; Wang et al., 2015; Colina et al., 2019; Wang et al., 2019; Zhang et al., 2020) have been extensively studied. Among them, SnRK proteins play essential roles throughout plant life.

SnRK protein kinases contain a similar Ser/Thr kinase domain, conserved UBA, and the KA1 domains in SnRK1. Meanwhile, the osmotic stress-activated domain I was found in SnRK2, and a unique NAF domain in the SnRK3 subfamily (Coello, Hey & Halford, 2011). Furthermore, some SnRK2 protein kinases have an acidic amino acid–base sequence called domain II that can participate in abscisic acid (ABA)mediated responses to abiotic stresses (Yoshida et al., 2006). The SnRK1 subfamily is evolved in early eukaryotes, before the divergence of fungi, animals, and plants (Halford & Hey, 2009). Thus, SnRK1 protein kinases in plants are highly homologous to SNF1 genes in yeast and AMP-activated protein kinases in mammals, which is mainly involved in carbon and nitrogen response metabolism and energy-sensing (Coello, Hey & Halford, 2011). In *Arabidopsis thaliana*, *AKIN10* and *IDD8* constitute a sugar metabolic pathway that mediates flowering time under low-sugar conditions (Jeong et al., 2015). Unlike the SnRK1 subfamily, SnRK2 and SnRK3 are unique in plants and are considered to be evolved from the SnRK1 family via gene duplication during plant evolution, playing a key role in the stress, calcium and ABA signaling pathway with epigenetic and metabolic responses (Halford & Hey, 2009). SnRK2 is the most widely studied subfamily and mainly focused on the participation of SnRK2 protein kinases in ABA-dependent and ABA-independent abiotic stress. In *A. thaliana*, 8/10 AtSnRK2 (Boudsocq, Barbier-Brygoo & Laurière, 2004; Boudsocq et al., 2007) and in *Oryza sativa*, all 10 stress/ABA-activated Serine/threonine-protein kinase 1 (OsSAPK1 to OsSAPK10) (Kobayashi et al., 2004) can be activated by hyperosmotic and saline stress. Among them, AtSnRK2.2/2.3/2.6 and OsSAPK8/9/10 were strongly activated by ABA and act as core positive regulators to regulate ABA-dependent stress responses (Fujita et al., 2009). The SnRK2 subfamily also plays a key role in the regulation of gene expression via activating basic region-leucine zipper (bZIP) transcription factors connected to an epigenetic mechanism that controls the activation or repression of a gene (Baena-González & Sheen, 2008; Fujii, Verslues & Zhu, 2011). The SnRK3 subfamily commonly called calcineurin B-like interacting protein kinases (CIPK) with a self-inhibitory NAF domain that can interact with calcineurin B-like (CBL) protein (Cheong et al., 2007). The CBL-CIPK protein complex constitutes a precise calcium signaling system, which plays a vital role in the process of achieving information regarding integration and physiological coordination to resist various stresses in plants (Tang et al., 2020; Tripathi et al., 2009). In *Arabidopsis*, *AtCIPK1* can interact with *CBL1* to participate in the ABA-independent signaling pathway and interact with *CBL9* in response to ABA-dependent pathways (D’Angelo et al., 2006). Furthermore, numerous shreds of evidence indicate that the SnRK family is widely involved in almost all hormone signaling pathways. For example, SnRK1 phosphorylates FUSCA3 (FUS3) and MYC transcription factors regulate ABA, ethylene, gibberellin synthesis and jasmonic acid signaling (Gazzarrini et al., 2004; Lumba et al., 2012; Im et al., 2014; Chan et al., 2017). SnRK2.8 phosphorylation NONEXPRESSER OF PATHOGENESIS-RELATED GENES1 (NPR1) respond to the systemic immunity in SA-independent systemic signals (Lee et al., 2015). In short, hormone signals are important for the metabolism of floral aromas and SnRK proteins are deeply involved in the hormone signaling pathway (Mai, Wang & Yang, 2011; Ma et al., 2018; Ke et al., 2019). However, either SnRK protein kinases respond to plant hormone signals or participates in the regulation of floral fragrance in *H. coronarium* is still unknown.

*H. coronarium* is a perennial herb, commonly known as “White Butterfly Flower” or “Butterfly Ginger”. *H. coronarium* is popular due to its elegant shape and refreshing fragrance of flower and widely cultivated in tropical and subtropical regions (Chen et al., 2013; Yue, Yu & Fan, 2014). The blooming of flower results in a strong refreshing scent which is mainly composed of monoterpenes, sesquiterpenes, and some benzenoids (Lan et al., 2013; Yue, Yu & Fan, 2015; Chen et al., 2019; Ke et al., 2019). Some studies also reported that hormone signaling plays an important role in floral scent formation (Schmelz et al., 2003; Dudareva et al., 2013; Cna’ani et al., 2015). SnRK gene family plays an essential role in plants, however, its function in floral scent formation is completely missing. Our previous research showed that auxin and auxin signaling components can alter the amount of floral volatile compounds (Ke et al., 2019). In the present study, a total of 60 *Hc SnRK* genes were identified and analyzed in *H*. *coronarium* genome. The expression patterns of *HcSnRK* gene family in response to several hormones (ABA, auxin, jasmonic acid and ethylene) and their corresponding hormone inhibitors were measured by qRT-PCR. Furthermore, based on the expression profile, two *HcSnRK* genes (*HcSnRK2.2* and *HcSnRK2.9*) were screened out and their involvement in the metabolism of floral fragrance was demonstrated by virus-induced gene silencing (VIGS). Also, the subcellular localization of these genes was performed. These results will provide the theoretical basis for better understanding the function of *SnRK* genes in hormone signaling and the regulatory metabolism of floral scent formation in *H*. *coronarium*.

## Materials and Methods

### Plant growth environment and hormone treatments

The plant materials were grown in a greenhouse under natural light (South China Agricultural University, Guangzhou, China). Plant material was immediately frozen in liquid nitrogen and stored at −80 °C for RNA isolation and further experimentation.

Plants used for hormone treatment were cut into about 40 cm with a wedge-shape and placed in a 500 mL beaker filled with ultrapure water. The concentration of hormones used for treatments was as followed: 400 µM for ABA, 100 µM for nordihydroguaiaretic acid (NDGA), auxin (IAA), methyl jasmonate (MeJA) and acetylsalicylic acid (ASA), 1.5 mM for 2-(4-chlorophenoxy)-isobutyric acid (PCIB), 10 µL/L for ethylene (ET), and 4 µL/L for 1-methylcyclopropene (1-MCP).

### RNA extraction, cDNA synthesis and qRT-PCR analysis

Total RNA was extracted using the HiPure plant RNA mini kit (Magen, China) according to the manufacturer’s suggestions. One microgram of total RNA was reverse transcribed using PrimeScript™ RT reagent Kit with gDNA Eraser (TaKaRa, Japan) according to the manufacturer’s instructions. The qRT-PCR experiment was executed in an ABI 7500 Fast Real-Time PCR System (Applied Biosystems, Waltham, MA, USA) by using iTaq™ Universal SYBR Green Supermix (BIO-RAD, Hercules, CA, USA) with a 20 µL sample volume according to the manufacturer’s protocols. The reaction system was as followed: 95 °C for 1 min, then 40 cycles of 95 °C for 15 s, 55 °C for 30 s, and 72 °C for 30 s. The relative expression level of each gene was calculated by the standard 2^−ΔΔCt^ method (Livak & Schmittgen, 2001).

### Sequence retrieval and genome-wide identification of *HcSnRK* genes

*Arabidopsis* protein sequences were obtained from the Phytozome database (http://www.phytozome.net/) (Lamesch et al., 2012), genome data of rice was downloaded from the Rice Annotation Project (RAP) (https://rapdb.dna.affrc.go.jp/) (Sakai et al., 2013), and genomic data (Supplementary raw data) of *H. coronarium* was obtained from the Beijing Novogene Bioinformatics Technology Corporation (China).

The local BLASTP search (E-value-5) was performed using the 39 and 48 SnRK protein sequences of *Arabidopsis* and rice according to the Hidden Markov Models profile from the Pfam database (http://pfam.xfam.org/) (Finn et al., 2016). The candidate HcSnRK protein sequences were sent to the NCBI Conserved Domain Database (http://www.ncbi.nlm.nih.gov/Structure/cdd/wrpsb.cgi) (Marchler-Bauer et al., 2011), SMART database (http://smart.embl-heidelberg.de/) (Letunic, Doerks & Bork, 2012) and Pfam database (Finn et al., 2016) for domain search. Based on the information from the above three databases, we manually select *HcSnRK* genes with conserved functional domains. Furthermore, molecular weight (MW) and isoelectric point (*pI*) of confirmed 60 HcSnRK protein sequences was calculated by ExPASy online software (http://www.expasy.ch/tools/pi_tool.html).

### Multiple sequence alignment and phylogenetic analysis of HcSnRKs

The ClustalX software (Thompson et al., 1997) was used for multiple sequence alignment of 60 HcSnRK protein and MEGA 7 software (Kumar et al., 2018) was used to construct a phylogenetic tree using the neighbor-joining method (Saitou & Nei, 1987) with 1,000 replicates of bootstrap values. The DNAMAN software was used to show multiple sequence alignment of 60 *HcSnRK* genes.

### Conserved motifs identification and gene structure analysis of HcSnRK

The exon-intron structure of the *HcSnRK* genes was performed using the Gene Structure Display Server (http://gsds.gao-lab.org/) (Hu et al., 2015) online program. The conserved motifs of HcSnRK protein sequences were identified by Multiple Expectation Maximization for Motif Elicitation (MEME) online software (http://meme-suite.org/tools/meme) (Bailey et al., 2009) with the following parameters: zero or one occurrence per sequence, 20 motifs should MEME find.

### Ka and Ks calculation and selection mode analysis

The ratio of non-synonymous substitutions (Ka) and synonymous substitutions (Ks) were used to analyze the selection modes of *HcSnRK* genes. Protein sequences without stop codon of HcSnRK were aligned by MEGA 7. The Ka and Ks values were calculated by DnaSP v5 software (Librado & Rozas, 2009) with following parameters: assign coding regions, from start to end; assign genetic code, nuclear universal.

### *Cis*-elements analysis *HcSnRK* genes

The upstream sequences 2000 bp of each *HcSnRK* gene were submitted to PlantCARE Database (http://bioinformatics.psb.ugent.be/webtools/plantcare/html/) (Lescot et al., 2002) to predict the function of *HcSnRK* genes. Six hormone-related *cis*-elements including ABA-responsive, auxin-responsive, jasmonic acid-responsive, ethylene-responsive, salicylic acid-responsive, and gibberellin-responsive were identified. Four *cis*-acting elements involved in plant stress responses, such as defense and stress responses, low-temperature responses, drought responses, and wound responses were analyzed.

### Subcellular localization of HcSnRK2.2 and HcSnRK2.9

HcSnRK2.2 and HcSnRK2.9 were fused into the vector pEAQ-HT-GFP using the *Age* I enzyme followed by transformation into *Agrobacterium tumefaciens* (strain EHA105). The infection solution (OD_600_ = 0.6) activated by MES solution (10 mM MgCl2, 10 mM MES and 100 µM acetosyringone, pH = 5.6) was injected into the *Nicotiana benthamiana* leaves as described previously (Ke et al., 2019). Two to three days later the leaves were visualized using Leica DM RXA2 upright fluorescent microscope with 40 × 0.75 numerical aperture objective as explained previously study (Yue, Yu & Fan, 2014; Abbas et al., 2019). The primers are listed in Table S5.

### Headspace floral volatiles analysis

The whole flower was placed in a closed 250 mL glass bottle supplemented with an internal standard. Polydimethylsiloxane (PDMS) fiber was inserted into the bottle for 15 min to adsorb volatiles for 15 min followed by insertion into the gas chromatography-mass spectrometry (GC-MS) system as explained previously (Ke et al., 2019; Yue, Yu & Fan, 2014).

### Virus-induced gene silencing (VIGS)

The barley stripe mosaic virus (BSMV) system was successfully applied in *H. coronarium.* The BSMV-VIGS system which consists of pCaBS-α, pCaBS-β, and pCaBS-*γ* vectors was kindly provided by Professor Dawei Li (China Agricultural University). Linearization of pCaBSγ vector using *Apa* I endonuclease and about 300 bp specific base sequence of *HcSnRK2.2* and *HcSnRK2.9* were fused to the vector according to the protocol system (Yuan et al., 2011) and optimized by extending the connection time to 2 min. The solution contains a mixture of an equal proportion of pCaBS-α, pCaBS-β, and pCaBS-γ/*HcSnRK2.2*/*HcSnRK2.9* with a final OD_600_ of 0.5 to 0.6. The flowers at Stage S1 were dipped into the solution followed by vacuum infiltration at -0.8 atmosphere standard for 10 min. After vacuum infiltration, the flowers were immediately washed with sterilized water and were placed in an incubator with following conditions: 12 h day/night period at 16 °C for 5 days (Ke et al., 2019). The floral volatile contents were measured as described above, and the experiment was repeated three to four times.

## Results

### Identification of *HcSnRK* gene family in *H. coronarium*

Base on the BLAST and Hidden Markov Model search, a total of 60 candidate genes were identified in *H. coronarium* genome. According to subfamily and chromosomal localization of genes, *HcSnRK* genes were named *HcSnRK1.1* ∼*HcSnRK1.4*, *HcSnRK2.1* ∼*HcSnRK2.13*, and *HcSnRK3.1* ∼*HcSnRK3.43*, respectively. The physical parameters of these genes are summarized in Table 1. The amino acid (aa) length ranged from 326 aa (*HcSnRK2.8*) to 526 aa (*HcSnRK3.14*), and the length of *HcSnRK2* subfamily is shorter than the other two subfamilies. The average length of *HcSnRK1*, *HcSnRK2*, and *HcSnRK3* subfamily were 500, 352, and 451 aa, respectively. Meanwhile, protein molecular weight varies greatly from 36.87 kDa (*HcSnRK2.8*) to 58.80 kDa (*HcSnRK3.14*), and the isoelectric point from 4.81 (*HcSnRK2.11*) to 9.48 (*HcSnRK3.12*).

**Table 1:** The characteristics of the *HcSnRK* gene family in *H. coronarium*.

Gene Name	Gene ID	Position	CDS (bp)	Amino Acids	Exons	*pI*	MW (kDa)
*HcSnRK1.1*	Hc42.68	Chr4:60322670-60328745(−)	1,497	498	11	8.43	56.70
*HcSnRK1.2*	Hc154.85	Chr14:13692396-13697801(−)	1,533	510	12	8.71	58.24
*HcSnRK1.3*	Hc25.126	Chr0:1124760-1129850(−)	1,497	498	11	8.63	56.16
*HcSnRK1.4*	Hc1223.4	Chr0:18406-24187(+)	1,482	493	12	7.08	40.51
*HcSnRK2.1*	Hc479.26	Chr5:3118456-3123025(+)	1,092	363	9	4.86	41.10
*HcSnRK2.2*	Hc163.43	Chr5:35295876-35298554(+)	1,098	365	9	5.48	41.76
*HcSnRK2.3*	Hc39.21	Chr5:45454971-45460547(+)	1,098	365	9	4.95	41.27
*HcSnRK2.4*	Hc782.12	Chr8:7883604-7886707(−)	1,125	374	10	6.00	43.00
*HcSnRK2.5*	Hc54.19	Chr8:40661379-40668342(−)	1,002	333	9	5.35	38.48
*HcSnRK2.6*	Hc843.18	Chr11:43521897-43525336(+)	1,020	339	9	5.67	38.47
*HcSnRK2.7*	Hc275.28	Chr13:2181732-2184478(+)	1,032	343	10	5.35	38.73
*HcSnRK2.8*	Hc326.23	Chr14:223114-225278(−)	981	326	9	5.26	36.87
*HcSnRK2.9*	Hc326.60	Chr14:473891-477474(+)	1,020	339	9	5.43	38.51
*HcSnRK2.10*	Hc57.125	Chr14:8431879-8435942(+)	1,092	363	9	4.95	41.08
*HcSnRK2.11*	Hc25.205	Chr0:1763461-1767704(+)	1,059	352	9	4.81	39.83
*HcSnRK2.12*	Hc191.34	Chr0:484266-493850(−)	1,077	358	9	5.80	41.17
*HcSnRK2.13*	Hc407.47	Chr0:377348-381541(+)	1,080	359	9	6.00	41.53
*HcSnRK3.1*	Hc430.44	Chr1:6617049-6620752(−)	1,452	483	2	6.56	54.12
*HcSnRK3.2*	Hc438.56	Chr1:11474898-11476860(+)	1,323	440	2	8.37	49.78
*HcSnRK3.3*	Hc438.54	Chr1:11603538-11605298(+)	1,314	437	1	8.96	48.32
*HcSnRK3.4*	Hc108.24	Chr1:28981635-28984540(+)	1,317	438	2	9.32	50.06
*HcSnRK3.5*	Hc253.154	Chr1:53838578-53842095(+)	1,377	458	12	8.58	51.54
*HcSnRK3.6*	Hc304.12	Chr2:35890951-35893118(+)	1,545	514	3	8.64	57.18
*HcSnRK3.7*	Hc219.55	Chr2:61929342-61931157(+)	1,338	445	1	7.31	49.19
*HcSnRK3.8*	Hc115.24	Chr3:6979416-6980852(−)	1,437	478	1	9.06	53.69
*HcSnRK3.9*	Hc171.23	Chr3:11928832-11930468(+)	1,236	411	1	9.31	44.60
*HcSnRK3.10*	Hc685.16	Chr3:24071137-24074279(+)	1,317	438	2	9.17	49.75
*HcSnRK3.11*	Hc55.127	Chr3:38306358-38309493(−)	1,341	446	14	8.94	50.68
*HcSnRK3.12*	Hc256.98	Chr4:8831260-8833000(−)	1,305	434	1	9.48	47.86
*HcSnRK3.13*	Hc280.50	Chr4:44679903-44683582(+)	1,332	443	3	8.75	50.59
*HcSnRK3.14*	Hc484.68	Chr5:184915-186806(+)	1,581	526	1	9.03	58.80
*HcSnRK3.15*	Hc484.67	Chr5:189058-191912(−)	1,401	466	3	8.45	52.34
*HcSnRK3.16*	Hc316.81	Chr5:44515665-44521645(−)	1,305	434	13	6.41	48.87
*HcSnRK3.17*	Hc32.21	Chr6:8904624-8906421(+)	1,356	451	1	8.80	50.82
*HcSnRK3.18*	Hc769.7	Chr6:51928707-51942543(+)	1,326	441	14	8.67	50.28
*HcSnRK3.19*	Hc3.374	Chr7:3703792-3708273(−)	1,365	454	14	5.66	51.08
*HcSnRK3.20*	Hc971.16	Chr7:7639489-7645951(+)	1,446	481	13	7.87	55.94
*HcSnRK3.21*	Hc33.46	Chr7:8729879-8733633(+)	1,311	436	2	9.06	49.17
*HcSnRK3.22*	Hc247.6	Chr8:47314082-47316537(−)	1,353	450	3	8.68	49.82
*HcSnRK3.23*	Hc102.99	Chr9:1148877-1153212(+)	1,323	440	16	6.82	49.96
*HcSnRK3.24*	Hc369.81	Chr9:2688438-2692404(+)	1,389	462	13	5.95	51.98
*HcSnRK3.25*	Hc71.72	Chr10:906032-912303(−)	1,134	377	14	5.78	42.95
*HcSnRK3.26*	Hc48.119	Chr11:9190841-9192641(−)	1,317	438	10	6.65	49.63
*HcSnRK3.27*	Hc439.14	Chr11:16527358-16532095(−)	1,386	461	15	8.70	52.46
*HcSnRK3.28*	Hc286.62	Chr11:46635686-46645148(−)	1,470	489	15	9.31	55.72
*HcSnRK3.29*	Hc158.15	Chr12:1277125-1278952(+)	1,449	482	2	6.15	53.97
*HcSnRK3.30*	Hc5.4	Chr12:34636145-34637497(+)	1,353	450	1	9.11	50.94
*HcSnRK3.31*	Hc79.41	Chr12:43896038-43897396(+)	1,359	452	1	6.74	50.06
*HcSnRK3.32*	Hc114.20	Chr14:2279142-2284718(−)	1,323	440	2	8.72	49.65
*HcSnRK3.33*	Hc279.1	Chr14:16032098-16047415(−)	1,332	443	8	8.75	50.19
*HcSnRK3.34*	Hc132.10	Chr15:33199307-33201621(+)	1,476	491	10	8.11	54.23
*HcSnRK3.35*	Hc74.133	Chr16:2385971-2387422(−)	1,452	483	1	6.11	54.51
*HcSnRK3.36*	Hc747.7	Chr16:36843545-36847614(+)	1,335	444	2	9.01	50.38
*HcSnRK3.37*	Hc26.164	Chr17:1572756-1575773(−)	1,308	435	2	9.16	49.33
*HcSnRK3.38*	Hc259.23	Chr17:6843287-6866867(−)	1,335	444	16	8.70	49.91
*HcSnRK3.39*	Hc259.98	Chr17:7354852-7357698(+)	1,392	463	13	7.22	51.51
*HcSnRK3.40*	Hc15.312	Chr0:2384884-2386204(−)	1,215	404	2	9.37	44.25
*HcSnRK3.41*	Hc15.375	Chr0:2948868-2950520(+)	1,446	481	1	8.88	53.95
*HcSnRK3.42*	Hc414.48	Chr0:563909-565482(+)	1,308	435	1	8.81	47.59
*HcSnRK3.43*	Hc444.50	Chr0:650222-651310(+)	1,089	362	1	7.01	40.66

### Phylogeny and multiple sequence alignment of *HcSnRK* gene family

**Figure 1:**
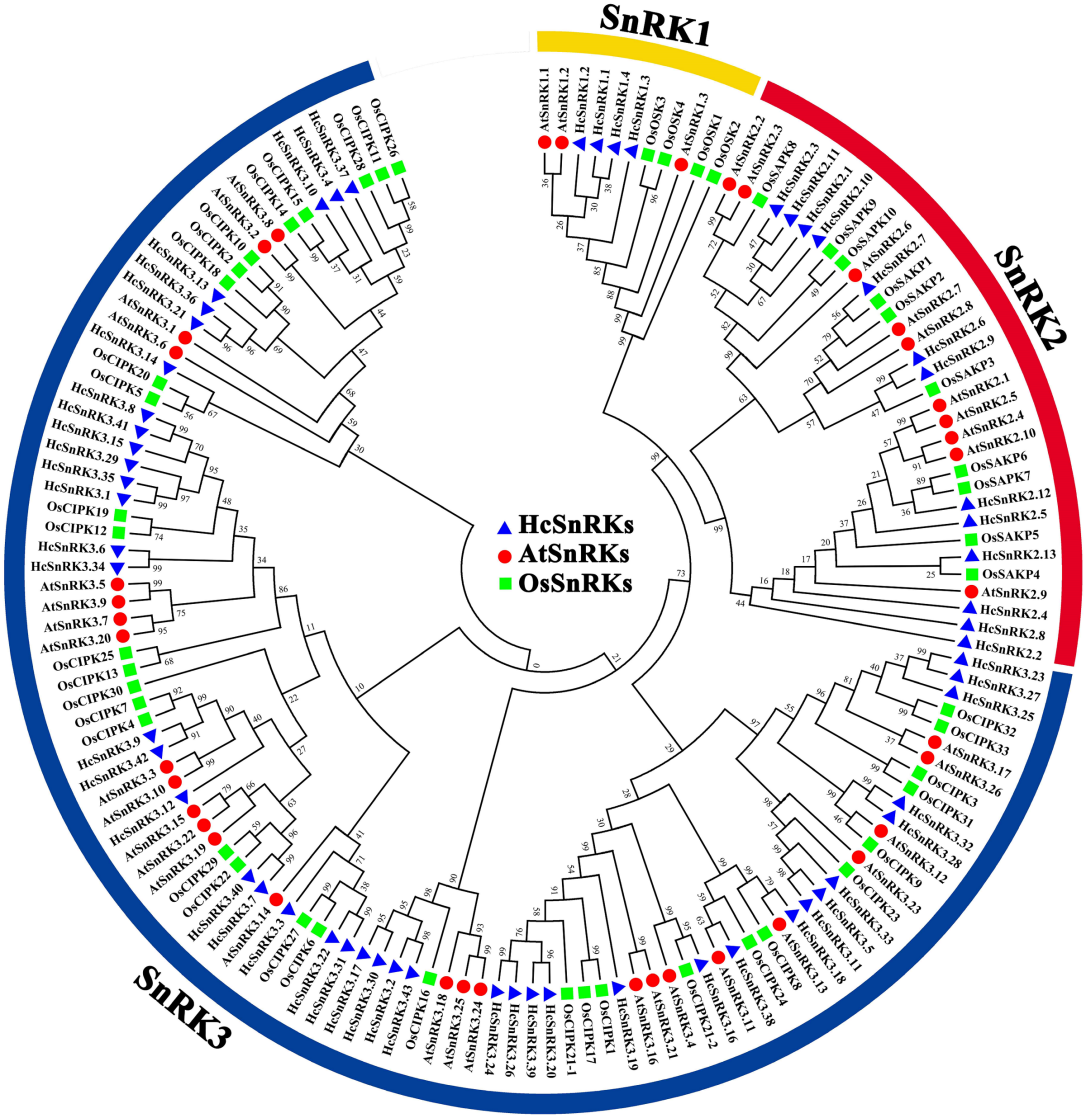
Phylogenetic tree of SnRK genes from *H. coronarium*, Arabidopsis, and rice. Sixty *HcSnRK* genes, 39 *AtSnRK* genes, and 48 *OsSnRK* genes are clustered as SnRK1, SnRK2, and SnRK3 subgroups. The detailed information of these genes from different plants are provided in Table S4. Clustal X software was used for multiple sequence alignment and MEGA 7 software used to construct a phylogenetic tree by the neighbor-joining method.

The evolutionary relationships of the *SnRK* genes in *H. coronarium*, *A. thaliana,* and *O. sativa* was revealed by constructing the phylogenetic tree based on multiple sequence alignment of amino acids. The full-length protein sequence of 39 AtSnRK, 48 OsSnRK, and 60 HcSnRK genes were used to construct a phylogenetic tree using MEGA 7 and by choosing the neighbor-joining method (Fig. 1). The results showed that 60 *HcSnRK* genes were divided into three groups as expected. Alike *Arabidopsis* and rice, the member of HcSnRK3 family were the highest (43) followed by HcSnRK2 (13) and HcSnRK1 (4), respectively.

**Figure 2:**
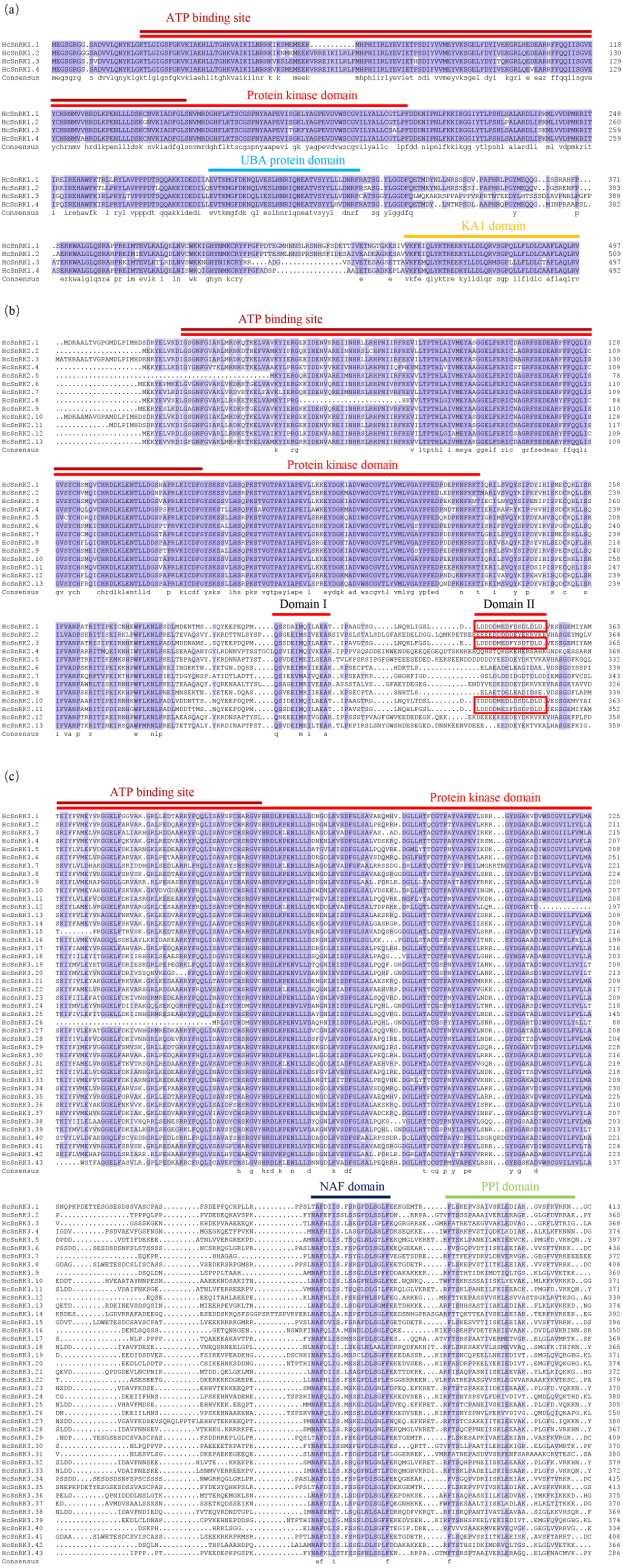
MSA of SnRK genes in *H. coronarium*. Sequences were aligned using Clustal W software and edited in Photoshop.

To explore the gene structure of the *HcSnRK* gene family, multiple sequence alignment was performed using DNAMAN 8.0 software. As shown in Fig. 2, ATP binding site and protein kinase active-site were found at N-terminal, and domain I was identified at C- terminal of *HcSnRK2* subfamily. Meanwhile, the amino acid sequence of the complete protein kinase domain and NAF domain were recognized in *HcSnRK3* subfamily. In summary, conserved domain analysis and multiple sequence alignment validated that all 60 *HcSnRK* genes have complete functional domains.

### Gene structural analysis of *HcSnRK* gene family

**Figure 3:**
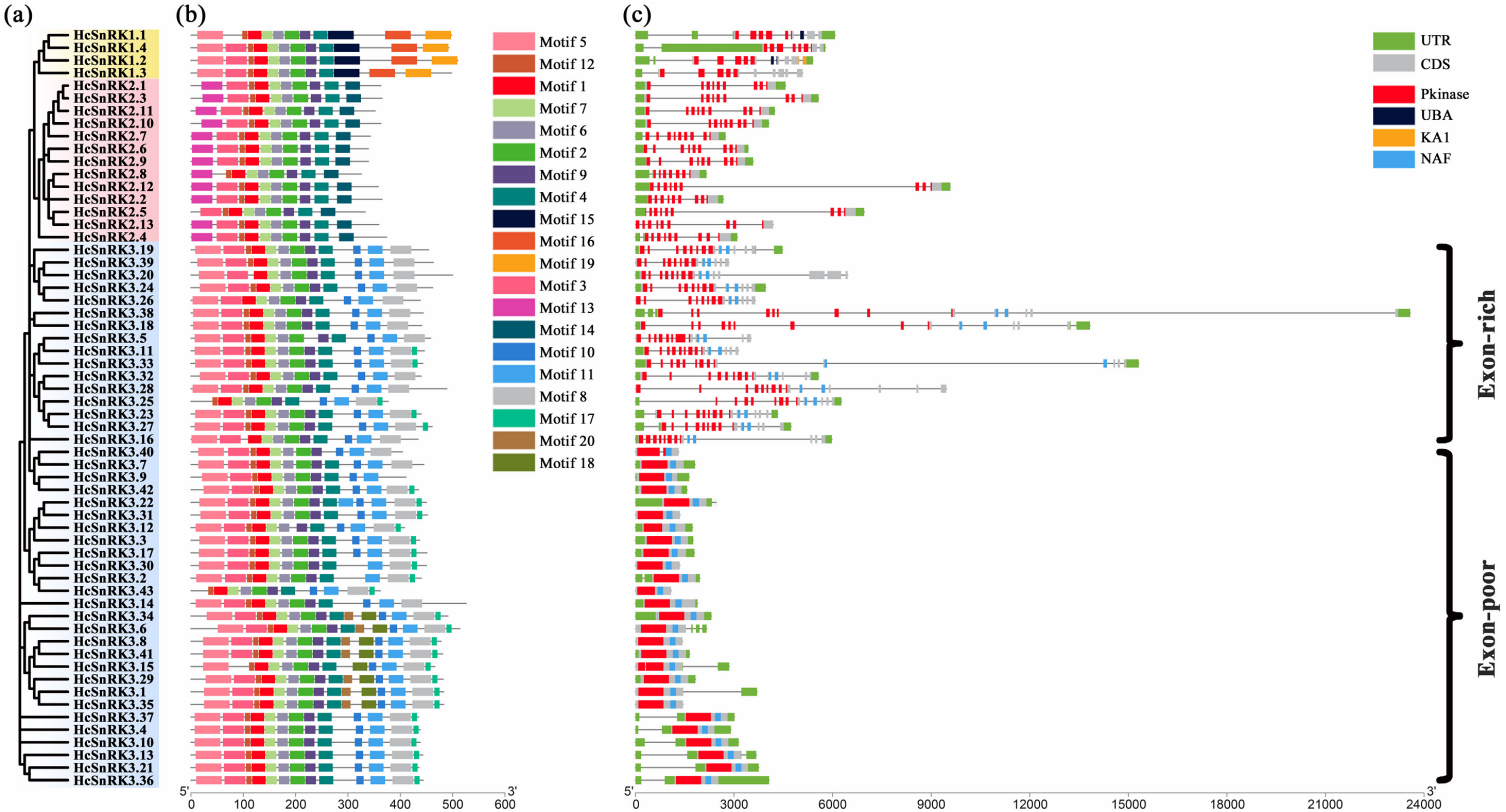
Phylogenetic relationships, conserved motifs, and gene structures of *HcSnRK* genes. (A) The phylogenetic tree of 60 *HcSnRK* genes was built via choosing the NJ method. Yellow, red, and blue background color represents SnRK1, SnRK2, and SnRK3 subgroups. (B) The conserved motifs were analyzed by MEME software. (C) Gene structure and conserved domains of SnRK genes.

The sequence structure of 60 HcSnRK proteins was analyzed using the MEME program by choosing the 20 motifs with default parameters (Table S1). The results showed that motif 1 encoded a protein kinase domain and was present in all *HcSnRK* genes, while different subfamilies retained an obvious difference in motif composition (Figs. 3A and 3B). Furthermore, motifs 1, 2, 6, 7, and 9 were present in all *HcSnRK* genes. Meanwhile, motif 19 was only found in the *HcSnRK1* subfamily which encoded the KA1 domain, whereas, motif 10 and 11 only appeared in the *HcSnRK3* subfamily, which encoded the NAF domain. Moreover, *HcSnRK* genes in the same subfamily have a similar motif, indicating that those genes have the same gene structure and functional domain. As for other motifs, the Pfam database did not find any functional annotations.

The combined phylogenetic tree and web server Gene Structure Display Server (GSDS) analysis was performed to determine the intron/exon structure of *HcSnRK* genes (Fig. 3C). The data showed that the members of the same subfamily share similar features. The *HcSnRK1* subfamily genes have 11 to 12 introns, while the *HcSnRK2* subfamily contains 9 to 10 introns. However, the number of introns in the *HcSnRK3* subfamily varies. The 27 *HcSnRK3* genes contained less than 3 introns, and the 16 *HcSnRK3* genes contained 8 to 13 introns. Therefore, *HcSnRK3* genes can be divided into two subgroups, intron-rich and intron-poor subgroups, respectively, based on the number of introns. Previously, similar intron numbers and classification pattern was observed in both monocots and dicots species, such as *Arabidopsis*, rice, maize, poplar, etc. The characteristic of intron number indicates that the evolution of *SnRK* genes was conserved in plants.

### Chromosomal location and gene duplication analysis

**Figure 4:**
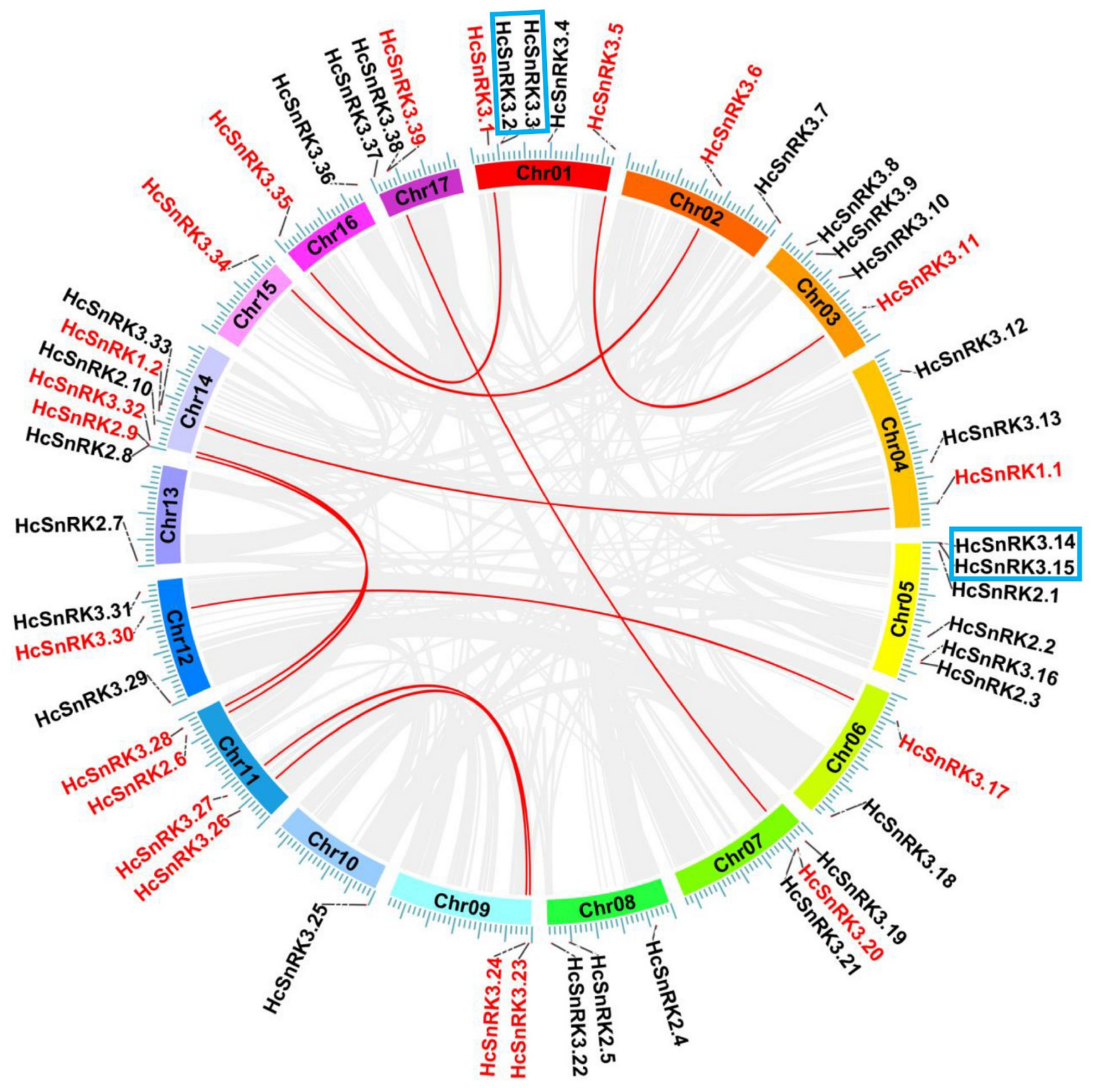
Chromosomal location and synteny analysis of *HcSnRK* in *H. coronarium* genome. *HcSnRK* genes are mapped onto all 17 chromosomes, gray background lines represent synteny blocks in whole *H. coronarium* genome and red lines showed the gene duplication pairs of *HcSnRK* genes.

The chromosomal localization analysis showed that 60 *HcSnRK* genes were unevenly mapped onto all 17 chromosomes, including four *HcSnRK1* genes, 13 *HcSnRK2* genes and 43 *HcSnRK3* (Fig. 4), whilst nine *HcSnRK* genes were localized on an unknown chromosome which will, later on, be assigned on anyone among 17 via refining whole-genome sequencing. Six *HcSnRK* genes were located on chromosome 5, and 14, while chromosome 1 has five *HcSnRK* genes distribution. However, chromosomes 10, 13, and 15 only contain one *HcSnRK* gene. In short, all the *HcSnRK* genes were randomly distributed on all chromosomes in *H. coronarium* genome.

To identify the segmental duplication of *HcSnRK* genes, BLAST and MCScanX methods were used. Among 60 *HcSnRK* genes, 10 duplicated pairs derived from segmental duplication and 2 from tandem duplication were observed (Fig. 4). The majority of the duplicated gene pairs were found on chromosome 11 and 14. To demonstrate and reveal the effect of selection pressure on the evolution of *HcSnRK* genes, synonymous (*Ks*), non-synonymous substitutions (*K*a), and *Ka*/*Ks* ratios per site between every duplicated pair were calculated by DnaSP 5.0 software. More importantly, *K*a/*K*s = 1 indicates neutral selection, *K*a/*K*s<1 represents purifying selection, and *K*a/*K*s*>* 1 represents positive selection and accelerated evolution. The *Ka*/*Ks* ratio of 12 duplicated gene pairs ranged from 0.095 to 0.465 (Table S2), suggesting that all duplicated gene pairs of *HcSnRK* had undergone purifying selection.

### *Cis*-regulatory elements analysis

**Figure 5:**
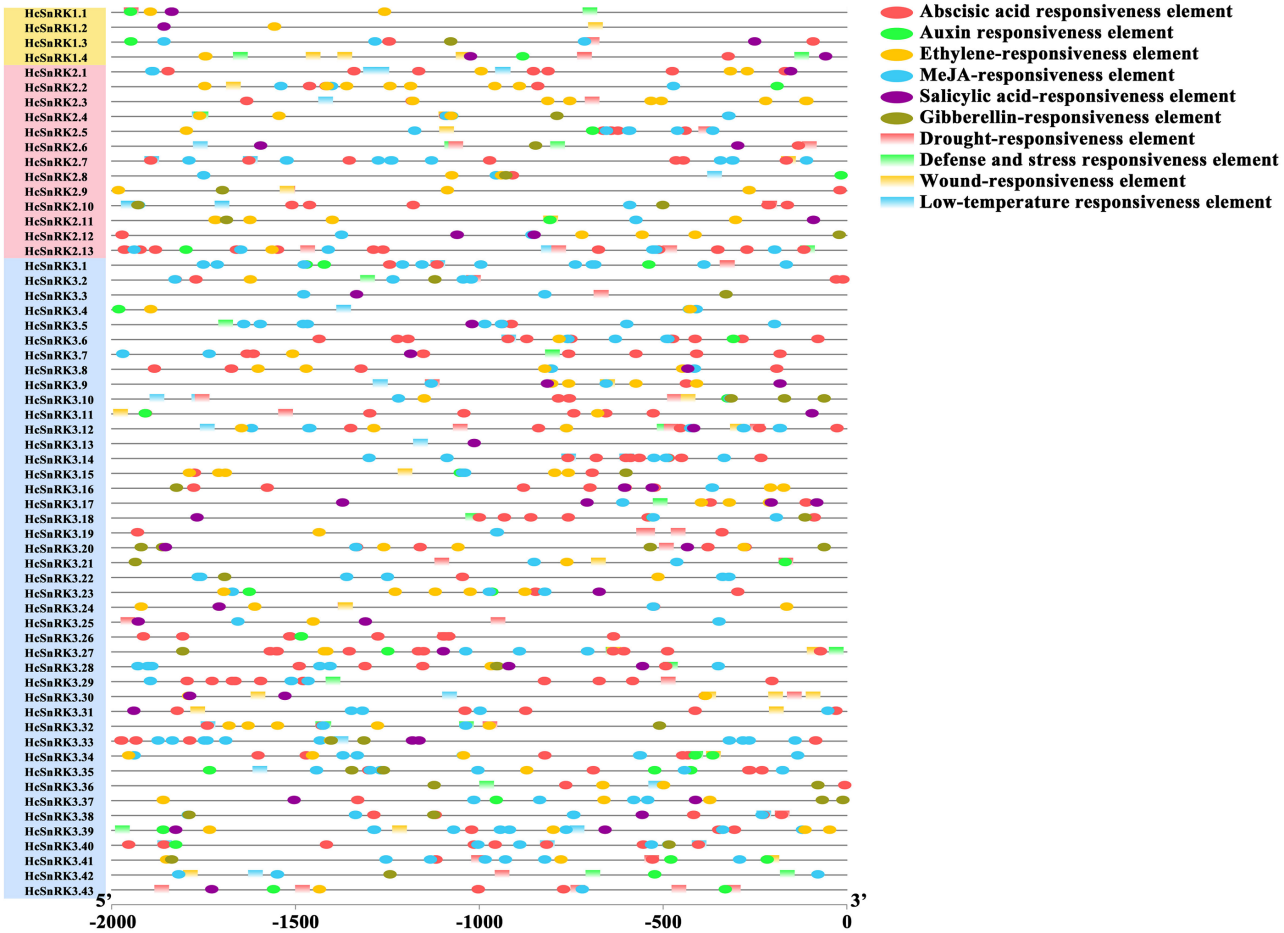
*Cis*-acting elements analysis of the *HcSnRK* genes promoter region. The 2,000 bp sequence before the start codon was used to analyze *cis*-acting elements. Different color boxes indicate different *cis*-acting elements.

The upstream 2 kb of promoter sequences of 60 *HcSnRK* genes were submitted to the PlantCARE database, to analyze the function and regulatory mechanism of *HcSnRK* genes. The results showed that almost all *HcSnRK* genes contained hormone-responsive *cis*-elements; however, less than half of *HcSnRK* genes contained abiotic stresses *cis*-elements. Interestingly, 53 out of 60 *HcSnRK* promoters contained ABRE *cis*-elements, while 50 and 44 out of 60 *HcSnRK* promoters contained MeJA-responsive and ethylene responsive *cis*-acting regulatory elements, respectively. Meanwhile, about half of the promoters of the *HcSnRK* gene contained auxin, salicylic acid, and gibberellin responsive *cis*-elements. On the other hand, less than half of the promoters of *HcSnRK* genes contained low-temperature and drought-inducible *cis*-elements, and about one-third of promoters contained defense, stresses, and wound-responsive element (Fig. 5; Table S3). *Cis*-regulatory element analysis data suggested that *HcSnRK* genes might respond to multiple hormones and abiotic stresses.

### Expression pattern of *HcSnRK* genes in different varieties, tissues and flower developmental stages

**Figure 6:**
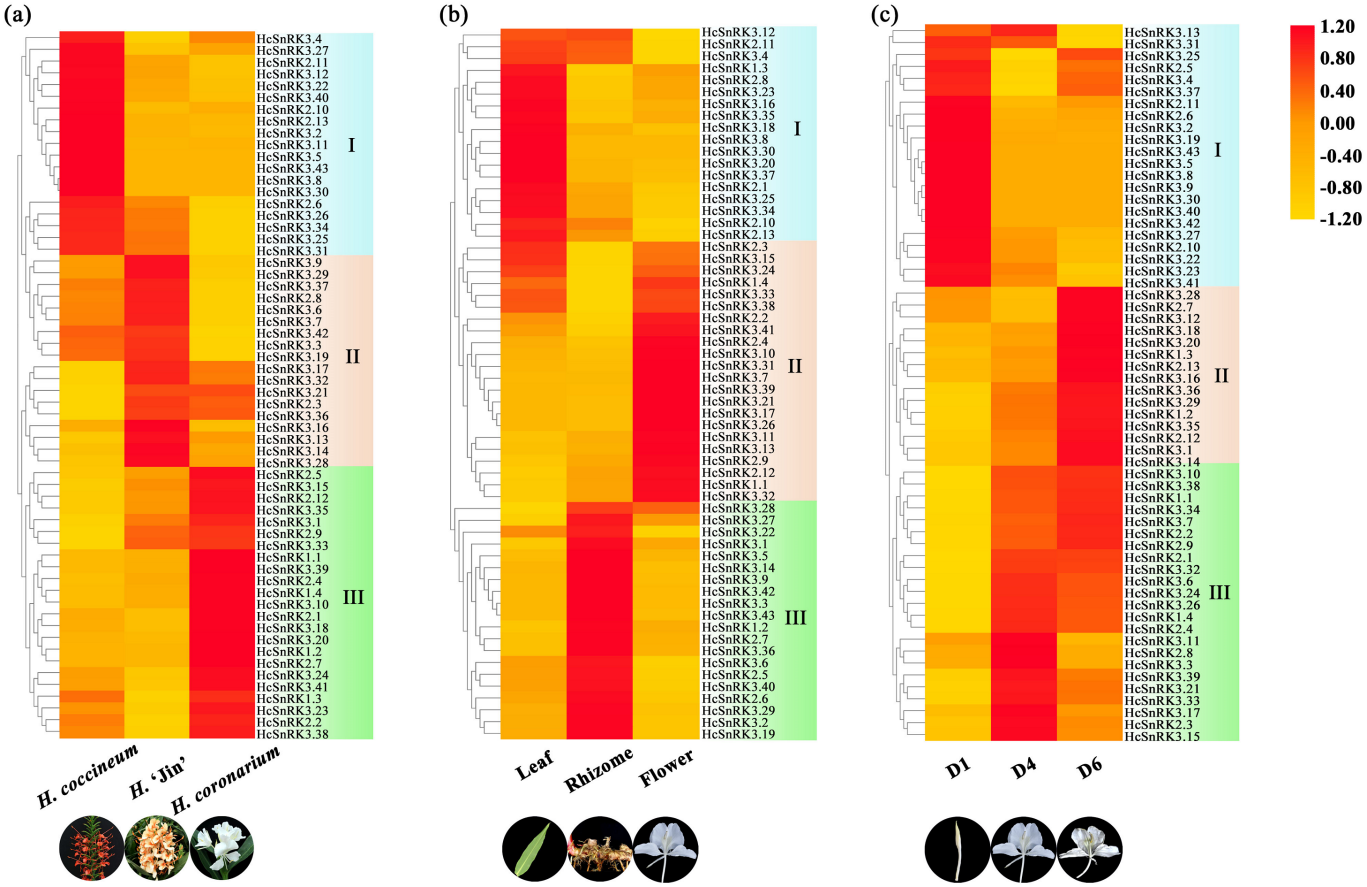
Expression Patterns of *HcSnRK* genes from different transcriptome data. (A) Heat map of *HcSnRK* genes expression pattern in three different varieties. (B) Expression patterns of *HcSnRK* genes in different tissues. (C) Expression profiles of *HcSnRK* genes in different flower developmental stages. Levels of upregulated expression (red) and downregulated expression (yellow) are shown on a log2 scale from the highest to lowest expression.

The analysis of differential expression pattern of *HcSnRK* genes was performed by using the transcriptome data of three different tissue (leaf, rhizome, and flower) and three flower development stages (D1; bud stage, D4; full-bloom and D6; flower senescence stage) of *H. coronarium* and three different varieties of *Hedychium* (Yue, Yu & Fan, 2015). The volatile compounds among different varieties vary significantly. The GC-MS analysis showed that the emission of volatile compounds from *H. coronarium* were higher as compared to *H. ‘Jin’*, while volatile compounds in *H. coccineum* are very low (Fan et al., 2007). The expression level of HcSnRK genes is presented in a heat map and HcSnRK genes with similar expression patterns were grouped into distinct groups. Cluster I represent the group of HcSnRK genes which had the highest expression in H. coccineum, Cluster II in * H. ‘Jin’* and cluster III had preferential expression *H. coronarium*, respectively (Fig. 6A). Previous studies showed that the number of volatile contents were higher in the flowers compared to leaf and rhizome. Moreover, the amount of floral volatiles was low at the D1 stage and peak at the full-bloom stage (D4) with flower development and declined at the D6 stage (Ke et al., 2019). Similarly, expression of *HcSnRK* genes in different tissues and different flower development stages of *H. coronarium* were also grouped in three clusters. The tissue-specific expression is important for gene functioning. Cluster I showed preferential expression in leaf, Cluster II represents the group of genes that had the highest expression in flower, and cluster III represents specific expression in the rhizome, respectively (Fig. 6B). Based on different transcriptome data and preferential expression of cluster III in *H. coronarium*, flower-specific expression and highest expression at full-bloom stage of flower indicate its potential role in the flower development and in the regulation of floral scent formation processes (Fig. 6C). Based on transcriptome data, *HcSnRK2.2* and *HcSnRK2.9* were screened out for further experimental analysis.

### Expression patterns of *HcSnRK* genes in response to hormone treatments

**Figure 7:**
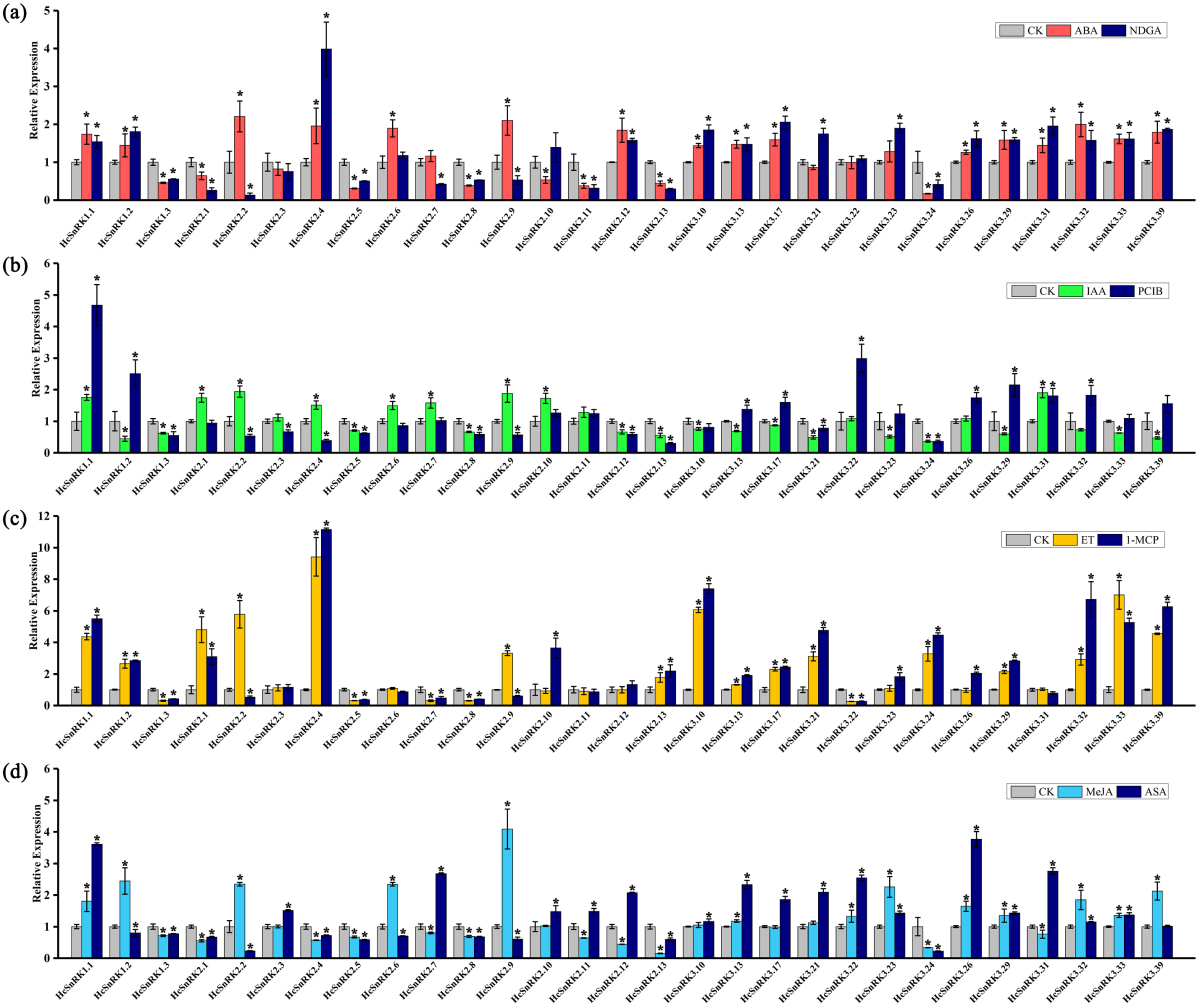
*HcSnRK* genes respond to several hormone treatments. The relative expression levels of *HcSnRK* genes in response to ABA (A), IAA (B), ET (C), MeJA (D), and corresponding inhibitor NDGA, PCIB, 1-MCP, AS was analyzed by qRT-PCR. The expression level of the control group was set to 1, error bars represent standard deviation from three to four biological replicates. Significant differences between the control group and hormone treatment samples are indicated by an asterisk (*p* < 0.05).

Auxin, ethylene, and ABA are the major hormones involved in the development of the flower. *H. coronarium* flowers were subjected to various hormone treatments. The results revealed that the volatile compounds of *H. coronarium* flowers were increased by 16%, 21%, 20%, and 22% under ABA, IAA, ethylene, and methyl jasmonate treatment, respectively. Meanwhile, the emission of volatile compounds decreased by 30%, 35%, 52%, and 34% under their corresponding hormone inhibitors nordihydroguaiaretic acid (NDGA), 2-(4-chlorophenoxy)-isobutyric acid (PCIB), 1-methyl cyclopropane (1-MCP) and acetylsalicylic acid (ASA), respectively (Ke et al., 2019). The expression level of selected *HcSnRK* genes was measured by qRT-PCR under hormone treatments (Fig. 7). The data showed that the expression level of *HcSnRK2.2* and *HcSnRK2.9* significantly up-regulated by ABA treatment, while down-regulated by NGDA (Fig. 7A). Overall, 24 *HcSnRK* genes were significantly up or downregulated under ABA treatment and 25 genes significantly changed under NDGA treatment. Under IAA and PCIB treatments, 24 *HcSnRK* genes showed significant difference in their expression pattern (Fig. 7B). Notably, *HcSnRK2.2*, *2.4,* and *2.9* genes significantly increased in IAA treatment and decreased in PCIB treatment. Furthermore, 21 *HcSnRK* genes significantly up or down-regulated after ethylene treatment, and 24 genes significantly changed after 1-MCP treatment (Fig. 7C). Moreover, *HcSnRK2.2* and *2.9* were highly up-regulated under ethylene treatment and down-regulated after 1-MCP treatment. In addition, 24 *HcSnRK* genes significantly increase or decrease in methyl jasmonate treatment, and 28 genes significantly changed in acetylsalicylic acid treatment (Fig. 7D). As expected *HcSnRK2.2*, *2.6,* and *2.9* genes significantly increased in methyl jasmonate treatment and decreased in acetylsalicylic acid treatment. In particular, the expression level of the *HcSnRK2.9* gene increased by 4 times under MeJA treatment. In short, plant hormones, such as ABA, IAA, ethylene, and jasmonic acid have a crucial effect on the regulation of floral aroma and the response pattern of *HcSnRK2.2* and *2.9* genes towards hormones were consistent with the changes of floral aroma contents. The results indicate that *HcSnRK2.2* and *2.9* genes play an important role in regulating the metabolism of floral aroma substances via crosstalk in hormone signaling.

### Subcellular localization of HcSnRK2.2 and HcSnRK2.9

**Figure 8:**
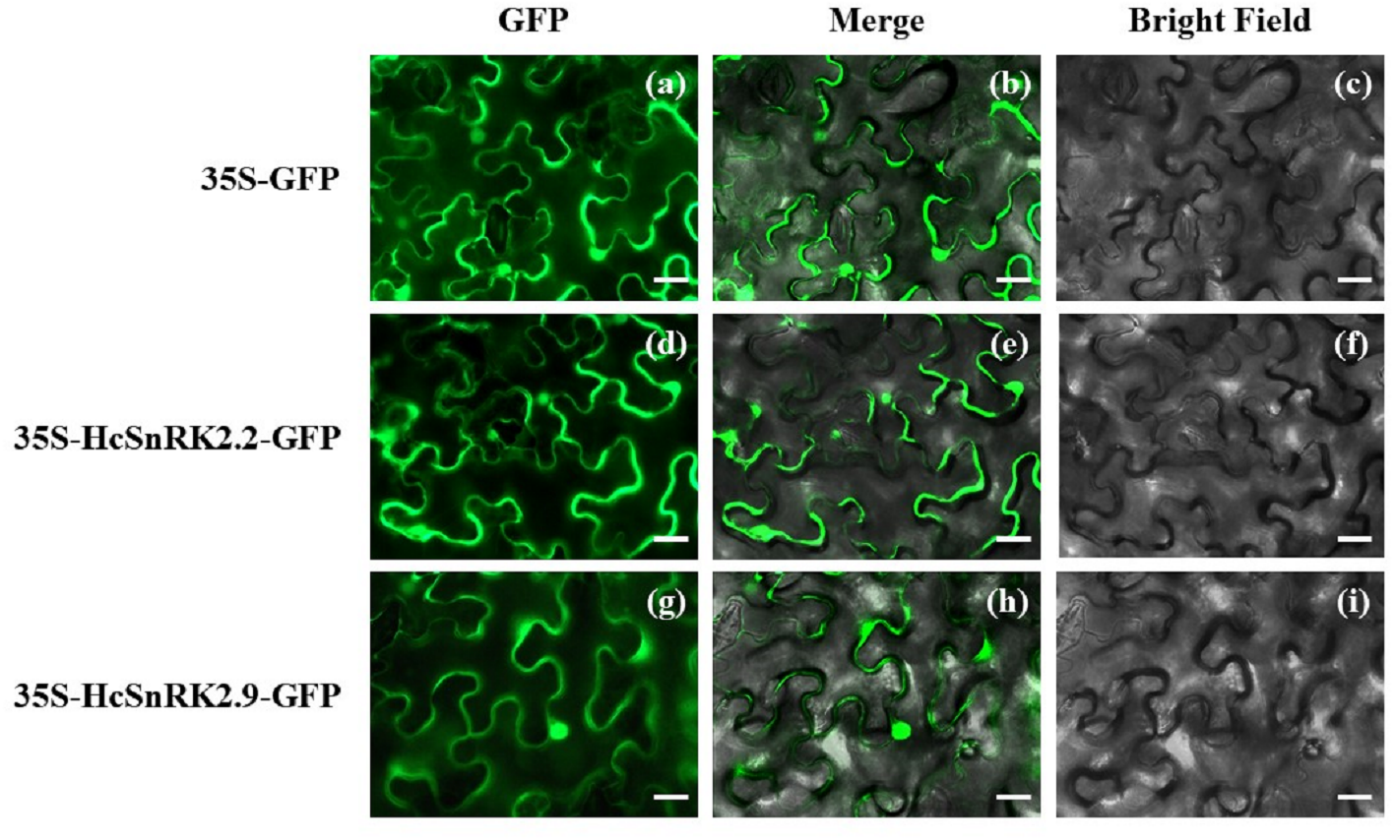
Subcellular localization of HcSnRK2.2 and HcSnRK2.9 proteins. HcSnRK2.2-GFP and HcSnRK2.9-GFP fusion vectors were transformed into *N. benthamiana* leaves and subcellular localization was carried out 48 h after infiltration using Leica DM RXA2 upright fluorescent microscope. The bar indicates 20 µm.

The amino acid sequence of HcSnRK2.2/2.9 was submitted to WoLP PSORT (https://wolfpsort.hgc.jp/) to predict subcellular localization. The predicted results showed that all HcSnRK2.2/2.9 proteins were expressed in the nucleus and cytoplasm. To experimentally verify the subcellular localization, full-length sequences of candidate HcSnRK2.2 and HcSnRK2.9 were fused to a GFP reporter gene and transferred to *N. benthamiana* leaves (Fig. 8). Subcellular localization experiments results revealed that both HcSnRK2*.2* and HcSnRK2*.9* were localized in the cell nucleus and cytoplasm as predicted. Similarly, multiple SnRK proteins have been reported previously from *Arabidopsis* and rice, which were in the nucleus and cytoplasm and were involved in the regulation of several ABA responses.

### Silencing of *HcSnRK2.2* and *HcSnRK2.9* genes

**Figure 9:**
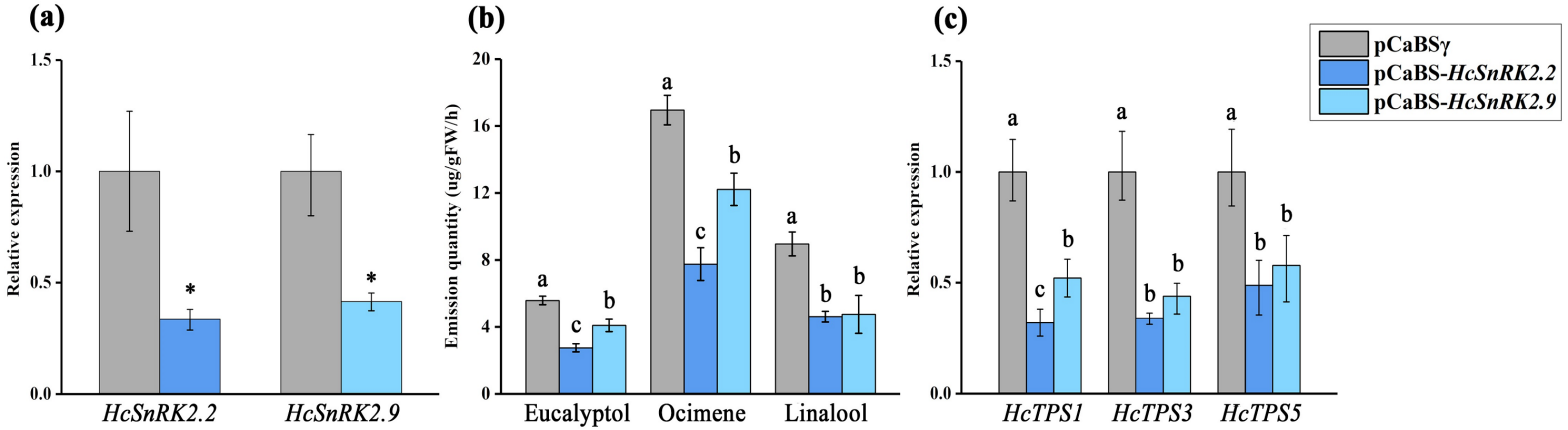
The silencing of *HcSnRK2*.2 and *HcSnRK2*.9 genes reduces the amount of the volatile compounds in *H. coronarium* flowers. (A) The relative expression level of *HcSnRK2*.2 and *HcSnRK2*.9 genes after BSMV-*HcSnRK2.2/2.9* silencing were analyzed by qRT-PCR. (B) The main volatile compounds measured by GC-MS after silencing of *HcSnRK2*.2 and *HcSnRK2*.9 genes. (C) The relative expression level of key volatile biosynthesis genes. Error bars represent standard deviations from three to four biological replicates and asterisks indicate statistically significant differences (*p* < 0.05).

To verify the function of *HcSnRK2.2* and *2.9* in the regulation of floral volatile synthesis, the virus-induced gene silencing (VIGS) system was used to suppress gene expression in *H. coronarium*. As shown in Fig. 9, the expression of *HcSnRK2.2* and *HcSnRK2.9* genes were significantly reduced by 66% and 58% compared to control, after silencing *HcSnRK2.2* and *2.9*, respectively. Moreover, the content of the main floral volatile substance, such as eucalyptol, ocimene, and linalool decreased significantly by 51%, 54%, and 48% after silencing *HcSnRK2.2* gene, and decreased by 27%, 28%, and 47% after silencing *HcSnRK2.9*, respectively. Furthermore, the expression levels of main volatile synthesis genes significantly decreased, such as *HcTPS1* which is responsible for eucalyptol synthesis, *HcTPS3* for ocimene, and *HcTPS5* for linalool synthesis. *HcTPS1*, *HcTPS3* and *HcTPS5* were down-regulated 68%, 66% and 51% after the suppression of *HcSnRK2.2*, and down-regulated 48%, 56% and 42% after silencing *HcSnRK2.9*. These findings indicate that *HcSnRK2.2* and *HcSnRK2.9* play an important key role in the regulation of floral aroma synthesis.

## Discussion

The *SnRK* gene family plays a crucial role in different physiological processes and is conserved in all eukaryotes. The *SnRK1* is involved in the functioning of cell energy sensing, while *SnRK2* and *SnRK3* play fundamental roles in the signaling pathway and the regulation of gene expression (Halford et al., 2004; Li et al., 2010; Colina et al., 2019). The *SnRK2* and *SnRK3* subfamily are unique in plants and originate from the duplication of the *SnRK1* subfamily (Colina et al., 2019; Halford & Hey, 2009). The expansion of the *SnRK* family may be partly since plants are sessile organisms and are forced to face more biotic and abiotic stresses than animals (Colina et al., 2019). In plants, the *SnRK* family represents an interface between stress signaling and metabolic pathway and is widely involved in ABA-dependent and ABA-independent abiotic stress. The *SnRK* family has been reported from a wide range of plant species, including *Arabidopsis*, maize (Chen et al., 2011), rice (Kanwar et al., 2014), and cotton (Cui et al., 2020), however, the *SnRK* family from *H. coronarium* has not been studied.

In the current study, 60 *HcSnRK* genes including 4 *HcSnRK1*, 13 *HcSnRK2,* and 43 *HcSnRK3* in *H. coronarium* were identified. Previously, 34, 48, 39, 44, and 52 *SnRK* genes have been identified form *Eucalyptus grandis* (Wang et al., 2019), *Oryza sativa* (Kobayashi et al., 2004), *A. thaliana* (Hrabak et al., 2003), *Brachypodium distachyon* (Wang et al., 2015) and *Glycine max* (Zhu et al., 2016), respectively. Moreover, different *SnRK* gene subfamily encompasses various conserved domains, however, all genes included a protein kinase domain present at the N-terminal. Phylogenetic analyses showed that like *Arabidopsis* and rice, *H. coronarium* also contain a similar number of *SnRK1* subfamily members (3 to 4) and *SnRK2* subfamily (10 or 13). However, the number of *SnRK3* subfamily genes varies from species to species, such as 26 in *A. thaliana*, 34 in *O. sativa*, 24 in *E. grandis*, 52 in *G. max,* and 43 in *H. coronarium*.

The different number of exon-intron also plays an important role in the evolution and function of a different gene family (Jo & Choi, 2015). The *SnRK3* subfamily not only varies on the number of genes, but previous findings also showed that the *SnRK3* subfamily can be subdivided into two clades according to the number of introns (Tang et al., 2016; Zhu et al., 2016). Likewise, the *HcSnRK3* subfamily can be subdivided into an intron-rich and intron-poor clade. The 16 *HcSnRK3* genes were grouped into an intron-rich clade (more than 8 introns) and 27 *HcSnRK3* genes in the intron-poor clade (less than 3 introns). Similarly, in *Arabidopsis* and rice, the *SnRK3* subfamily was subdivided according to the number of introns indicating that an increase or decrease in the number of introns can promote the structural evolution of the *SnRK3* gene family before eudicot–monocot divergence (Zhu et al., 2016). Recent findings suggest that the *SnRK3* subfamily originated in green algae, and the intron-poor group first appeared in the seed plants (Colina et al., 2019). It has been assumed that when seed plants will face great environmental pressure during evolution, intron-rich groups will lose intron and become intron-poor groups (Colina et al., 2019). Also, *HcSnRK1* has 11 to 12 introns, *HcSnRK2* subfamily has 9 or 10 introns. These results indicated that the number of introns in *HcSnRK* genes is similar to other plants. The conserved motif analysis revealed that gene structure and conserved motifs were similar in the same subfamily, indicating the close evolutionary relationship within the same subfamily, but different subfamilies involved in different stress response pathways.

A large number of *cis*-elements related to hormone response were found in the promoter sequences of *HcSnRK* genes, suggesting that *HcSnRK* genes respond to multiple hormone signals and interact with other metabolic pathways. Plants have developed unique strategies to cope with the external environment. Numerous evidences indicated that the *SnRK* family is widely involved in the response to various biotic and abiotic stresses, including salt, high or low temperature, and drought (Tang et al., 2016; Zhu et al., 2016; Wang et al., 2019). Many *cis*-elements related to stresses, wounding and defense response were identified in the promoter sequences of *HcSnRK* genes. Previous findings indicate that hormones play essential roles in the flower development and regulation of aroma (Chandler, 2011; Iqbal et al., 2017; Ke et al., 2019). The ethylene, Auxin, ABA, and MeJA responsive *cis*-elements were found in the majority of the promoters of *HcSnRK* genes suggesting their significant functions by crosstalk with *HcSnRK* genes in *H. coronarium* flower. The above results are in line with the previous findings from tomato and *H. coronarium* (Audran-Delalande et al., 2012; Ke et al., 2019). In our previous research, we describe that *Auxin*/*IAA* genes are involved in the regulation of floral scent and the volatile contents of *H. coronarium* flower were altered under different hormone treatments (Ke et al., 2019). Moreover, ethylene and ABA are also involved in floral scent regulation and flower senescence. To verify the response of the *HcSnRK* genes to several hormones, the expression levels of 29 genes based on their higher abundance in flower, was performed by qRT-PCR. The results showed that 27 *HcSnRK* genes significantly respond to ABA treatment, while 28, 24 and 28 *HcSnRK* genes significantly responded to IAA, ethylene, MeJA, and their corresponding inhibitors, respectively. Similarly, *HbSnRK2.5*, *2.7,* and *2.10* from *Hevea brasiliensis* were also significantly up-regulated under ABA, ethylene, and MeJA treatment. Alike, *HbSnRK2.8*, *2.9* up-regulated under ABA and MeJA treatment, whilst, *HbSnRK2.2* down-regulated under ABA and MeJA treatment, however, *HbSnRK2.6* significantly up-regulated under MeJA, down-regulated under ethylene and do not respond to ABA treatment. In the present study, *HcSnRK2.4* significantly up-regulated under IAA and down-regulated under PCIB, while *HcSnRK2.6* up-regulated under MeJA and down-regulated under ASA treatment. Furthermore, *HcSnRK2.2* and *HcSnRK2.9* showed significant differential expression under ABA, IAA, ethylene, MeJA, and their corresponding inhibitor treatments. Previous studies verified that ABA, IAA, ethylene, and MeJA have a significant effect on the regulation of floral aroma. These results implied that *HcSnRK2.2* and *HcSnRK2.9* maybe involved in multiple hormone metabolism pathways to regulate the metabolism of floral fragrance.

Tissue-specific expression pattern of *HcSnRK* genes in different tissue permits different regulation of tissue development and alternate means of metabolic regulation. The transcriptome data of different varieties (strong, moderate, and almost no floral fragrance variety), different tissues (rhizome, leaf, and flower), and different flower development stages (bud stage, full-bloom stage, and fade stage) were used to analyze the expression pattern of *HcSnRK* genes. The 23 *HcSnRK* genes showed high expression in strong floral fragrance variety, 22 *HcSnRK* genes were highly expressed in the flower, whereas, 23 *HcSnRK* genes showed their preferential expression at the full-bloom stage of the flower. It was also observed that 15 *HcSnRK* genes have extremely high expression during the senescence stage indicating their possible role in the regulation of flower aging. Moreover, the expression pattern of *HcSnRK2.2* and *HcSnRK2.9* was similar to the emission of floral substances, suggesting that these two genes might play an important role in the regulation of floral aroma synthesis. The differential expression pattern of *SnRK* genes has been found in many species. In apple, *MdCIPK4*, 9, 15, and 32 were highly expressed in the flower and *MdCIPK 29* show relatively high expression in fruit implied their different biological functions in respective tissues (Niu et al., 2018). In *Brassica napus* L., *BnCIPK9* was tissue-specific and developmental stage-specific expressed in seed, and overexpression of *BnCIPK9* reduced oil synthesis in the transgenic plant (Guo et al., 2018). The virus-induced gene silencing of *HcSnRK2.2* and *HcSnRK2.9* confirm their role in floral scent regulation. The silencing of *HcSnRK2.2* and *HcSnRK2.9* genes did not alter the flowering process, however, resulted in the emission of the low amount of floral volatile and decreases the expression pattern of key genes involved in the biosynthesis of floral scent (Fig. 9). The decrease in the emission of volatile compounds might be because of their involvement in the hormone signaling especially in ABA and ethylene signaling pathway. This is the first report regarding the role of *HcSnRK* genes in the regulation of the floral scent biosynthetic pathway. The function of *SnRK* family in ABA-dependent and independent pathway have been extensively studied, however, their role in floral scent pathway needs to be elucidated further.

## Conclusion

In brief, we identified the *SnRK* gene family in *Hedychium coronarium*; analyzed expression profiles based on three different transcriptome data, and screened numerous key candidate genes for functional characterization. Through virus-induced gene silencing, we find out the functional involvement of *HcSnRK2.2* and *HcSnRK2.9* in floral scent formation. Our findings will bring new insights into the function of *HcSnRK* genes in secondary metabolism

##  Supplemental Information

10.7717/peerj.10883/supp-1 Supplemental Information 1 Supplemental Tables **Table S1:** Sequences information of 20 *SnRK* Motifs.
**Table S2:**Ka/Ks ratios of paralogous *HcSnRK* genes.
**Table S3:** *Cis*-acting regulatory elements numbers found in the promoter region of *HcSnRK* genes.
**Table S4:** Gene IDs of *SnRK* genes from Arabidopsis and rice.
**Table S5:** List of primers used in the experimentations.

10.7717/peerj.10883/supp-2 Supplemental Information 2 *HcSnRK* gene information

10.7717/peerj.10883/supp-3 Supplemental Information 3 Raw data for Figures 7 & 9

## Abbreviations

SnRK: Snf1-Related protein kinase
VIGS: Virus-induced gene silencing
qRT-PCR: Quantitative real-time polymerase chain reaction
MAPK: Mitogen-activated protein kinase
CDPK: Calcium-dependent protein kinases
GSK3: Glycogen synthase kinase 3
SNF1: Sucrose non-fermenting 1
ABA: Abscisic acid
SAPK: Stress/ABA-activated protein kinase
bZIP: Basic region-leucine zipper
CIPK: Calcineurin B-like interacting protein kinases
NDGA: Nordihydroguaiaretic acid
IAA: Indole-3-Acetic Acid
PCIB: 2-(4-chlorophenoxy)-isobutyric acid
ET: Ethylene
1-MCP: 1-Methylcyclopropene
MeJA: Methyl jasmonate
ASA: Acetylsalicylic acid
GSDS: Gene Structure Display Server
MEME: Multiple Expectation Maximization for Motif Elicitation
Ka: Non-synonymous substitutions
Ks: Synonymous substitutions
PDMS: Polydimethylsiloxane
GC-MS: Gas chromatography-mass spectrometry
BSMV: Barley stripe mosaic virus
GFP: Green fluorescence protein

## Additional Information and Declarations

### Data Availability

The following information was supplied regarding data availability: Raw data are available in the Supplemental Files. Sequences available at GenBank: MW393703–MW393762.
